# Judicial analytics and the great transformation of American Law

**DOI:** 10.1007/s10506-018-9237-x

**Published:** 2018-12-10

**Authors:** Daniel L. Chen

**Affiliations:** Toulouse School of Economics, Institute for Advanced Study in Toulouse, University of Toulouse Capitole, Toulouse, France

**Keywords:** Judicial analytics, Causal inference, Behavioral judging

## Abstract

Predictive judicial analytics holds the promise of increasing efficiency and fairness of law. Judicial analytics can assess extra-legal factors that influence decisions. Behavioral anomalies in judicial decision-making offer an intuitive understanding of feature relevance, which can then be used for debiasing the law. A conceptual distinction between inter-judge disparities in predictions and inter-judge disparities in prediction accuracy suggests another normatively relevant criterion with regards to fairness. Predictive analytics can also be used in the first step of causal inference, where the features employed in the first step are exogenous to the case. Machine learning thus offers an approach to assess bias in the law and evaluate theories about the potential consequences of legal change.

## Introduction

Predictive judicial analytics holds the promise of increasing efficiency and fairness of law. Many talk of machine learning algorithms predicting decisions (Aletras et al. 2016) or even replacing judges (D’Amato 1976). But this article describes a set of findings showing that the decisions are not pure and can reflect bias (conscious and unconscious) and extra-legal factors such as time of day. This means that to predict decisions we will have to model factors which really should have no place in the decision making, so that accuracy is not always a good thing.

Consider a definition of justice as equal treatment before the law and equality based on recognition of difference. We can imagine a set of covariates *X* that should lead to the same prediction or predictability of outcomes $Y=f(X)+\varepsilon$; the *X*’s should improve predictions. And, we can think of a set of *W*’s that should not ($y\perp W,var(\varepsilon )\perp W$). We tend to think of *X*’s as mutable—as consequences of choices ($a\rightarrow X,a\nrightarrow W$), and the *W*’s as immutable, unrelated to one’s actions. These equations derive from the control principle (Moulin 2004; Gurdal et al. 2013), the idea that we are morally assessable only to the extent that what we are assessed for depends on factors under our control. Two people ought not to be morally assessed differently if the only other differences between them are due to factors beyond their control.

A highly predictive model would include the *W*. But, if we want to replace judges we should do so with machines that do not exhibit the biases and foibles. Since many think that highly accurate predictions relative to a large body of historic cases would provide a good indication that judges could be replaced, this article highlights the need to de-bias the predictions so the law could be applied without distortion by these extra-legal factors, which are enshrined in the earlier decisions—a single landmark case can overturn decades of decisions. Prediction is not a good measure of the accuracy of the model to what the law should be, since it will need to reflect biases and prejudices that ought to be excluded. Learning from large data can be used to identify these biases and prejudices. This article describes a number of findings indicating behavioral anomalies in judicial decision-making, which offers an intuitive understanding of feature relevance.

Inter-judge disparities in predictions ($Y=f_{j}(X)+\varepsilon$) is one salient example of a normative criteria with regards to fairness. Inter-judge disparities in prediction accuracy ($Y=f_{j}(X)+\varepsilon _{j}$) is as another. Not all behavioral anomalies can be detected, so the degree of susceptibility to unobserved behavioral anomalies would be captured by inter-judge disparities in prediction accuracy. Early predictability is yet another normative criteria with regards to fairness. If a judge can be predicted prior to observing the case facts, one might worry about the use of snap or pre-determined judgements, or judicial indifference. To put it differently, the preferences of judges over the legally relevant covariates may affect the influence of irrelevant features. A judge could be said to have weak preferences, meaning that there was a relatively low cost in departing from the legally optimal outcome. In such cases of legal indifference, irrelevant factors can be expected to have greater influence. Behavioral bias reveals when decision-makers are indifferent. Disparities in prediction accuracy can be called, difference in indifference.

Besides alerting to possible biases, machine learning algorithms can be used to evaluate the effects of the decisions. Just as much legal research makes recommendations (inputs) based on theories about the potential consequences of legal change, the predictions of decisions can be used for downstream analyses of causal evaluation of the effects of decisions. The predictions would not be used to suggest a decision, but used as inputs to increase efficiency and fairness of law. A causal inference framework is presented from where predictive analytics is used in the first step, where the features employed in prediction are exogenous to the case.

Counter-intuitively, a tension arises between uncovering bias and distortion to de-bias the law, and using predictions based on these biases to assess the consequences of law. If the last century of American law was characterized by what Karl Polanyi (1944) might call, “the great transformation^1^”, whereby American law was characterized by a shift to a consequentialist mode of reasoning about the law and a focus on efficiency, then judicial analytics might be the next step in this great transformation, to move from theorizing about the consequences of law to measuring the consequences of law and a focus on fairness.

Section 2 describes my findings on behavioral judging and judicial analytics. Section 3 discusses difference in judicial indifference. Section 4 shows how to measure causal impacts of judicial precedent. Section 5 concludes.

## Behavioral judging and judicial analytics

This article begins with describing briefly findings from other articles, which the reader can refer to for the theoretical and empirical details. These findings mostly serve as scene setters for using behavioral anomalies to predict judicial decisions. I use several exhibits from own work on the Circuit Courts, District Courts, the US Supreme Court, the immigration courts, and a district attorney’s office in New Orleans. Using machine learning to predict the legal decision raises the possibility of judicial analytics to uncover the factors that affect judicial decisions.

For those articles, I have digitized all 380,000 cases and a million judge votes from 1891 in the Circuit Courts. I have engineered 2 billion N-grams of up to length 8 and 5 million citation edges across cases, collated 250 biographical features on 268 judges, and linked this to the 5% random sample^2^ of over 400 hand-coded features and 6000 cases hand-coded for meaning in 25 legal areas. I also utilize a data set on millions of criminal sentencing decisions in US District Courts since 1992, linked to judge identity via FOIA-request, and a digital corpus of their opinions since 1923. These data are linked to publicly available Supreme Court datasets, US District docket datasets, geocoded judge seats, biographies of judicial clerks, and administrative data from Administrative Office of the US Courts (date of key milestones, e.g., oral arguments, when the last brief was filed, etc.) for measuring priming of identity, peer effects, perfectionism, partisan ways of persuasion, judicial innovation, and the genealogy of ideologies and schools of thought in temporally and spatially disaggregated text.

I have also the universe of administrative data on 1 million refugee asylum and 15 million hearing sessions and their time of day across 50 courthouses and 20 years (with randomly assigned judges) and hand-collected biographical data to study gambler’s fallacy, implicit egoism, habit formation, racial contrast, mood, extraneous factors, and time of day effects on judges’ normative commitments. I have a linked universe of individuals in a federal prosecutor’s office over a decade with many stages of random assignment, to measure, e.g., name letter effects, in-group bias, and the intersection of hierarchy and race. I have also digitized the speech patterns in US Supreme Court oral arguments since 1955—longitudinal data on speech intonation (linguistic turns) are rare. The data are linked to oral advocates’ biographies, faces, clipped identical introductory sentences, and ratings of their traits. The data are used to test labor market treatment of mutable characteristics and persuasion, and mimicry between lawyers and Justices and among Justices over time using high-dimensional econometrics.

These data serve as a natural laboratory to study normative judgments using the tools of machine learning and causal inference. Each setting offers unique features to study behavioral judging (see Table 1). The federal courts offer a setting to study the causal effects of common law precedent. The various possible anomalies explored are suggested by theories, mainly in economics and psychology, which are discussed in the original papers and reports consolidated here.

**Table 1:** Influences on judicial decisions

Circuit	District	SCOTUS	Asylum	New Orleans DA
Priming	Mood	Masculinity	Gambler’s fallacy	Implicit egoism
Deontological	Interpellation	Mimicry	Snap judgments	Hierarchy
Economics	Economics	Visual cues	Mood/time	Judge versus prosecutor

**Fig. 1:**
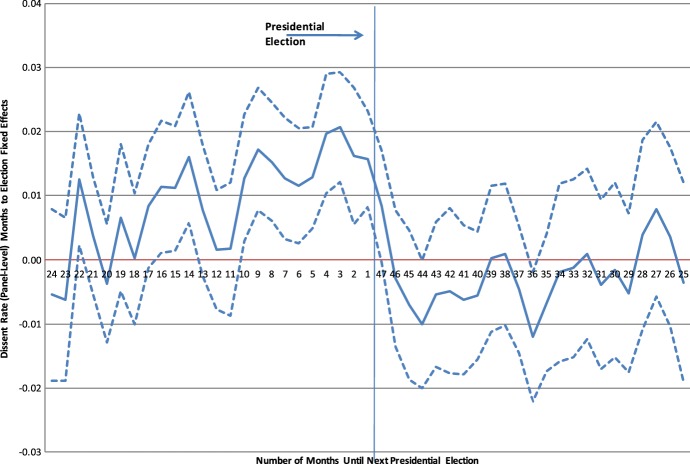
Electoral cycles among US courts of appeals judges. *Source*: Berdejo and Chen (2017)

Berdejo and Chen (2017) and Chen (2018) document how Circuit Court judges’ behavior varies over the Presidential election cycle. In particular, dissents (2-1 decisions) systematically increase before a Presidential election as shown in Fig. 1. This figure plots the monthly dissent rate relative to the month after the election. The solid line indicates the point estimates and the dotted lines the confidence intervals.

To get a sense of the magnitude, we might expect that on a three-judge panel, when you have both Democrats and Republicans appointees being assigned to the same panel, dissents are more likely. Indeed, as Table 2 shows, cases are 1.5% points more likely to have a dissent. However, when it is the quarter before the election, there is an additional 5–6% points greater likelihood in having a dissent. These effects are quite large, relative to the average rate of dissent, which is 8%. The table presents a linear regression of the probability of a dissent on a set of dummy indicators for each quarter prior to an election (the omitted quarter is the one after an election). The different columns present different sets of additional controls and a probit model instead of a linear probability model.

**Table 2:** Electoral cycles among US courts of appeals judges (regression). *Source*: Berdejo and Chen (2017)

	Dissent (2-1 decision) with or without dissenting opinion			
	Ordinary least squares		Probit	
	(1)	(2)	(3)	(4)
Divided (DRR or RDD)	0.0157	0.0153	0.114	0.111
	[0.00452]***	[0.00451]***	[0.0327]***	[0.0328]***
Quartertoelect = 1	0.0637	0.0527	0.448	0.377
	[0.0123]***	[0.0132]***	[0.0857]***	[0.0936]***
Quartertoelect = 2	0.0347	0.0255	0.284	0.224
	[0.0121]***	[0.0138]*	[0.0960]***	[0.105]**
Quartertoelect = 3	0.0325	0.0302	0.270	0.256
	[0.0123]***	[0.0134]**	[0.0982]***	[0.103]**

Standard errors in brackets (* *p* < 0.10; ** *p* < 0.05; *** *p* < 0.01)

**Fig. 2:**
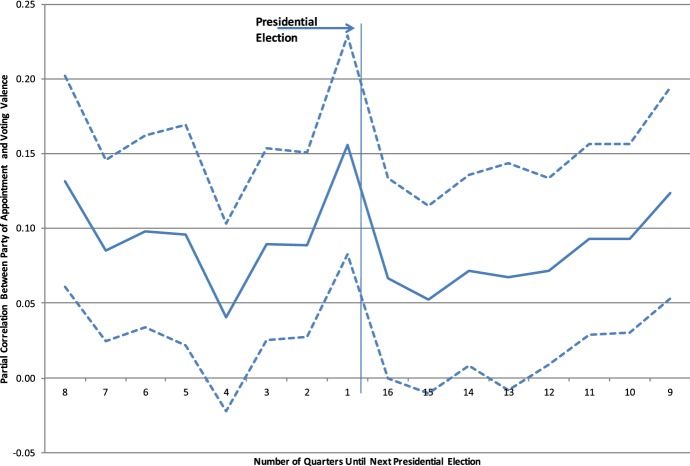
Electoral cycles in partisan voting. *Source*: Berdejo and Chen (2017)

Electoral cycles can be seen not only in dissent, but also in how judges vote. The 5% sample codes by hand each vote as conservative or liberal. Figure 2 shows that Democrats do vote more liberally relative to Republicans, but the correlation increases before the Presidential election. This figure is based on a regression of the vote valence on a set of dummy indicators and their interaction between party of appointment. The figure plots the coefficients of the interaction terms.

Electoral cycles also appear in legal precedent. Restricting to the sample of cases decided by unified panels (panels composed of three Republicans or three Democrats), Table 3 shows there is an increase in the correlation between the party of appointment and the valence of the precedent. This table presents a regression of the vote valence on the party of appointment interacted with a dummy indicator for the quarter before an election. If we think that precedent dictating a liberal or conservative outcome should be equally likely to appear for the different types of panels, then this result would suggest that the common law is being affected by these electoral cycles.

**Table 3:** Impact on precedent. *Source*: Berdejo and Chen (2017)

	Liberal precedent			
*Panel B: Politically unified panels (DDD or RRR)*				
Lastquarter	− 0.194	− 0.282	− 0.225	− 0.325
	[0.105]*	[0.154]*	[0.164]	[0.161]**
Appointed by Democrat	0.163	0.232	0.217	0.247
	[0.0303]***	[0.0423]***	[0.0468]***	[0.0447]***
Appointed by Democrat	0.208	0.288	0.237	0.345
*Lastquarter	[0.126]*	[0.178]	[0.193]	[0.183]*
Controls	Y	Y	Y	Y
Observations	5659	5659	5659	5659
R-squared	0.100			

Standard errors in brackets (* *p* < 0.10; ** *p* < 0.05; *** *p* < 0.01)

The impact of Presidential elections is further supported by Table 4, which shows that the Circuit Courts are also changing how they affirm or reverse the District Courts. This table presents a regression of the affirm or reverse decision on the quarter before an election including the controls listed.

**Table 4:** Electoral cycles in treatment of lower courts. *Source*: Berdejo and Chen (2017)

	(1)	(2)
	Affirm	Reverse
Mean of dep. var.	0.568	0.269
Last quarter	− 0.0588**	0.0519***
	(0.0251)	(0.0166)
Year FE	Yes	Yes
Circuit FE	Yes	Yes
Season FE	Yes	Yes
Legal issue FE	Yes	Yes
Divided (RDD or DRR) FE	Yes	Yes
Quarter-to-election FE	Yes	Yes
Observations	18686	18686
R-squared	0.054	0.025

Standard errors in parentheses (* *p* < 0.10; ** *p* < 0.05; *** *p* < 0.01)

**Fig. 3:**
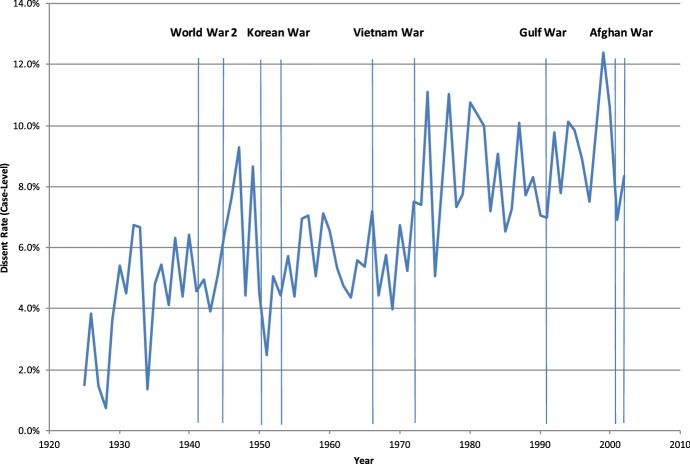
Effect of wartime on dissents. *Source*: Berdejo and Chen (2017)

We have seen that presidential elections polarize federal appellate judges to increase dissent, partisanship of precedent, and reverse lower courts. Chen (2018) documents polarizing effects that vary by intensity of elections across states, within judges, and over the electoral season. Within the timeline of a case, the electoral cycle only appears using the publication date, consistent with a transient priming mechanism. The effect appears largest on cases involving economic activity, a topic made salient during the election season. If elections spur partisan identities, during a period of national reconciliation, we would expect the opposite. Figure 3 shows that judges are less likely to dissent. This figure plots the mean dissent rate for each year. The vertical bars indicate the official start and end dates of wars.

In a second judicial anomaly, I explore the gambler’s fallacy, a well-known tendency for individuals to misunderstand random processes. In a series of coin flips, they think that there is a relative rapid alternation between heads and tails. But a real sequence of coin flips can reflect streaks of 1’s and streaks of 0’s. In other words, any two sequences will be equally likely, but judges may think a sequence like 0101001011001010100110100 is more likely than a sequence like 0101011111011000001001101. A judge granting asylum may worry about being too lenient if s/he grants too many decisions in a row or being too harsh if s/he denies too many in a row. Such a judge might actively, negatively autocorrelate. Indeed, if the previous decision was to grant asylum, the next decision is 1–2% points less likely to grant asylum. Table 5 presents a regression of the current decision on the lag decision including the controls listed. This effect is also observed in other situations where decision-makers make judgments, like with loan officers and baseball umpires (Chen et al. 2016b).

**Table 5:** Gambler’s fallacy in asylum decisions. *Source*: Chen et al. (2016b)

Dependent variable	Grant		
	(1)	(2)	(3)
Lagged grant	− 0.0159***	− 0.0116***	− 0.0156***
	(0.00422)	(0.00401)	(0.00422)
Applicant controls	Yes	Yes	Yes
Num prev asylums granted by judge	Yes	Yes	Yes
Num prev asylums granted in city	Yes	Yes	Yes
Judge-specific time trends	No	Yes	No
Time of day	No	No	Yes
N	106071	106071	106071
R^2^	0.125	0.167	0.126

Standard errors in parentheses (* *p* < 0.10; ** *p* < 0.05; *** *p* < 0.01)

**Fig. 4:**
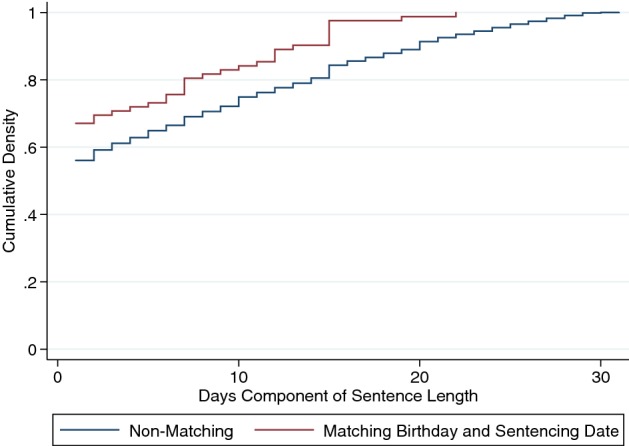
Judicial leniency on defendant birthdays in district courts (CDF). *Source*: Chen and Philippe (2017)

Mental accounting is the idea that we have mental categories. We have money for books or money for restaurants. When it comes to judges making sentencing decisions, they may have a category for sentencing months and one for sentencing days. Chen and Philippe (2017) finds that when it is the defendant’s birthday, judges round down in the number of sentencing days. Figure 4 plots the cumulative distribution function of non-zero days for sentences that occur on the defendant’s birthday and for those that do not.

**Fig. 5:**
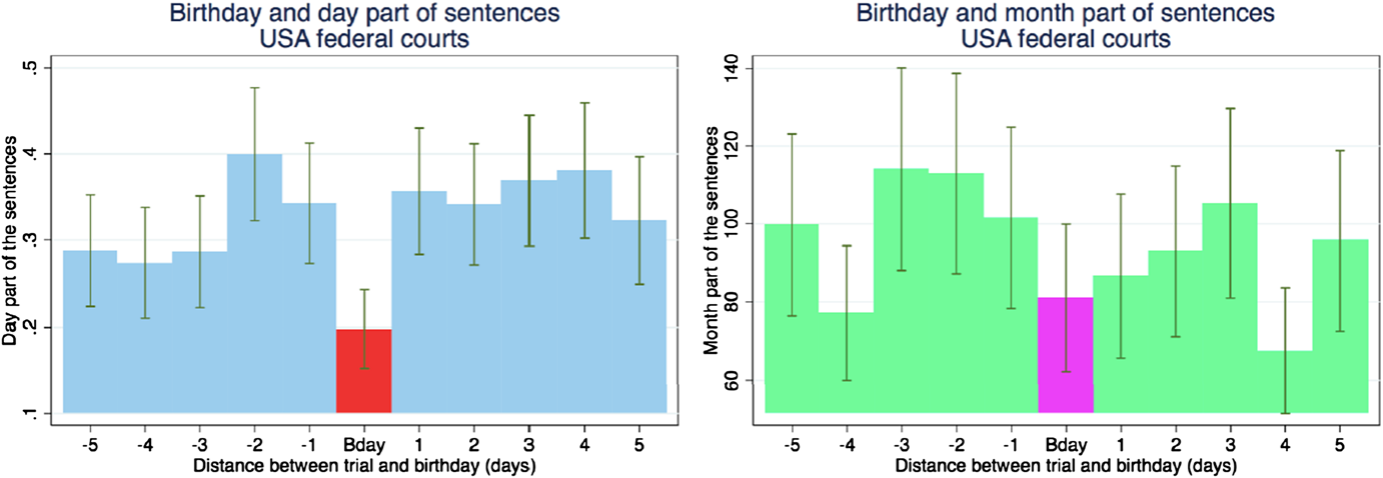
Judicial leniency on defendant birthdays in district courts. *Source*: Chen and Philippe (2017)

This effect is quite substantial and is only on the day of the birthday, not for days before or after, and it is not observed for sentencing months. Figure 5 presents the means for the days before and after a birthday and shows no effect on the months component of sentences.

**Fig. 6:**
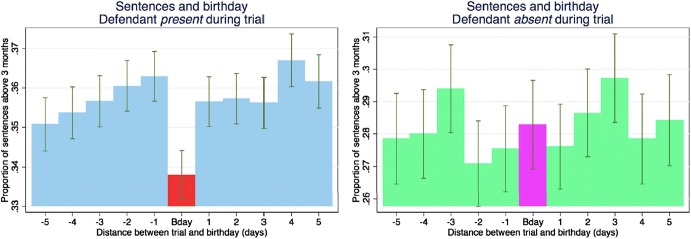
Judicial leniency on defendant birthdays in french courts. *Source*: Chen and Philippe (2017)

For French courts, where the defendants are not always present, the birthday effect is only observed when the defendant is present. Figure 6 presents the means for the days before and after a birthday for defendants who are present and for those who are not present. The norm in France is to appear at trial.

**Fig. 7:**
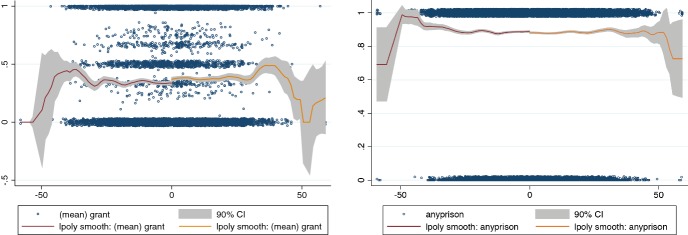
Mood in asylum and district courts. *Source*: Chen (2017)

A recent study by Eren and Mocan (2018) finds that Louisiana judges respond to the Louisiana football team winning or losing. Figure 7 shows the same effect in asylum courts and district courts with a much larger sample. The lines are local polynomials estimated for wins and for losses separately, the shaded area indicate the confidence interval, and the dots are jittered plots of the underlying data.

Notably, the effect in asylum courts comes entirely when the lawyer is not present. Table 6 presents a regression with an interaction between the lawyer being present and whether the NFL football game resulted in a win or loss with the controls listed. The first coefficient indicates that a win increases the chances of an asylum grant by 3.7% points relative to when there is a loss, but if there is a lawyer present, the effect essentially disappears.

**Table 6:** Effect of NFL outcomes by lawyer representation. *Source*: Chen (2017)

Dependent variable	Granted asylum	
	(1)	(2)
Yesterday’s NFL Win	0.037**	0.037**
	(0.014)	(0.014)
Yesterday’s NFL Win X	− 0.032*	− 0.032*
Lawyer	(0.017)	(0.017)
Lawyer	0.186***	0.186***
	(0.022)	(0.022)
JudgeXCity fixed effects	Yes	Yes
Time control	City-specific trends	City-specific trends
Week fixed effects	Yes	Yes
Season fixed effects	Yes	Yes
Applicant controls	Yes	Yes
N	22282	22282
${\rm R}^{2}$	0.30	0.30
Clustering	City	City+Judge
Number of clusters	56 × 340	56 × 340

Standard errors in parentheses (* *p* < 0.10; ** *p* < 0.05; *** *p* < 0.01)

**Fig. 8:**
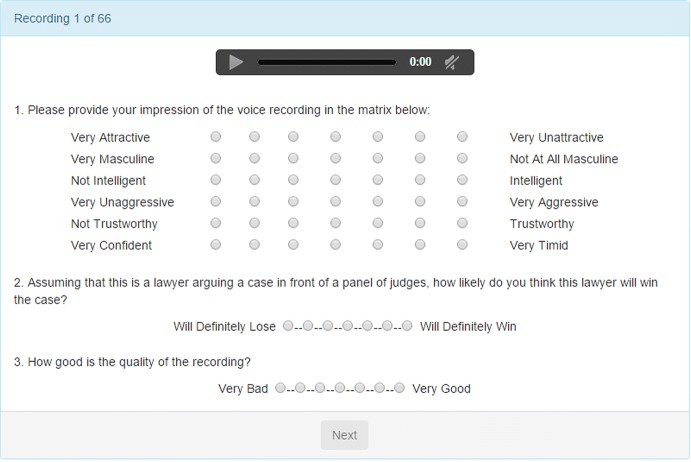
Questionnaire. *Source*: Chen et al. (2016a, 2017a)

So with the Supreme Court, I have been working with the oral arguments in two different ways. First, I have been clipping the first sentence, which is identical for all the lawyers—“Mr. Chief Justice, may it please the Court”—and asked third-party raters to rate the voices on attractiveness, masculinity, intelligence, and so on. Figure 8 shows a sample questionnaire used for the 1901 US Supreme Court oral arguments between 1999 and 2013.

**Fig. 9:**
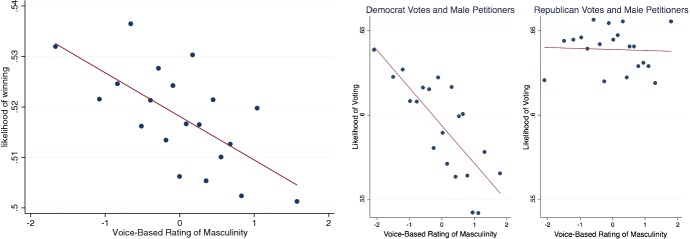
Supreme court votes and voice masculinity. *Source*: Chen et al. (2016a, 2017a)

Figure 9 shows that perceived masculinity predicts court outcomes. Males are more likely to win when they are perceived as less masculine. This figure presents a binscatter where each dot presents the mean x- and y-value for every 5% of the data along the x-axis. The first plot shows the overall relationship, which is then broken by the party of the judge. The line is a linear regression fit. The negative correlation between masculinity and win rates appears to be due to two mechanisms. First, the votes of Democrats and not Republicans are negatively associated with perceived masculinity.

**Fig. 10:**
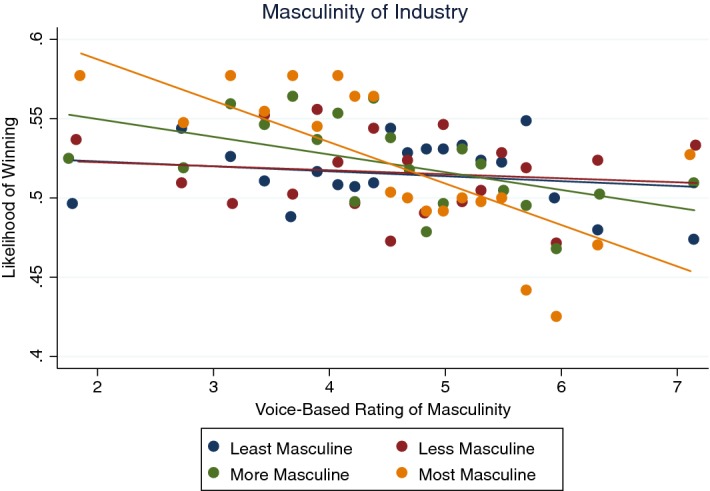
Masculinity of industry and response to masculinity. *Source*: Chen et al. (2017a)

Second, the relationship seems due to industry. Figure 10 shows that the correlation is stronger in more masculine industries. Each line or set of dots is presenting the relationship for quartiles of industry by masculinity rating. The category for industry comes the hand-labeled category of the parties involved in the litigation. We conceptualize three layers of actors: the judges, the lawyers, and the law firms who select the lawyers. A law firm that misperceives the masculine lawyer as being more likely to win or, prefers masculine lawyers for non-economic reasons, may choose more masculine lawyers at the cost of winning. The preference for more masculine lawyers may be stronger in more masculine industries.

I also align the audio with the text so we can extract the way each vowel is spoken to investigate a number of behavioral hypotheses. For example, it turns out that linguistic convergence is predictive of how judge decide (Chen and Yu 2016). Everyone also converges, lawyers to judges and judges to lawyers. This convergence can also be called mimicry.

**Fig. 11:**
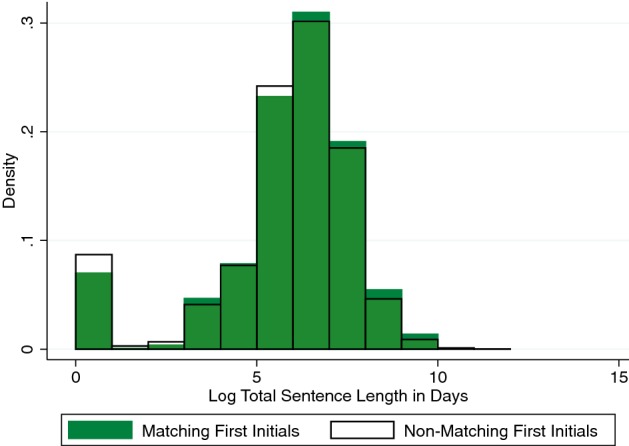
Name letter effects in sentencing decisions. *Source*: Chen (2016)

In psychology, people have documented that people respond differently when the first initial of their name is shown in the lab. This method related to implicit egoism has been used in many different experiments. In this setting, the defendants and the judges’ names are available, and Fig. 11 shows that when the first initials match, there’s an effect of matching on first initials on sentencing decisions. This figure presents the density of sentences for defendants whose first initial matches the first initial of the judge. It overlays the density of sentences for defendants whose first initials do not match the first initial of the judge. Fewer sentences of 0 and 1 years are assigned when the first initials match.

What this amounts to is 8% longer sentences when judges match on first initials with the defendant. The effect is consistent with self-image motivations to create social distance from negatively-valenced targets perceived to be associated with the self. The effects are larger for black defendants classified (by police) as “Negro” rather than “Black”. The first initial effect replicates for the last name, as does the difference by racial label. These results are robust to adjusting for controls including skin, hair, and eye color. Name letter effects appear for roughly all judges and amplify when the first and second letter of the name match, when the full name matches, or when the name letter is rare.

## Machine learning and judicial indifference

A prominent American jurist, Jerome Frank, proposed that “uniquely individual factors often are more important causes of judgments than anything which could be described as political, economic, or moral biases” (Hutcheson and Joseph 1929; Frank 1930 [2009])). This view is often caricatured as “what the judge had for breakfast” (Schauer 2009). The previous section has shown a collection of judicial anomalies, but these are findings using only data that already exists. Since a judge can be influenced by many factors unobserved to the statistician, an open question is how to assess the other unobserved influences in aggregate. Together, the psychological, political, economic, and moral biases that lead decisions of one judge to differ from another may be captured in unpredictability, the $\varepsilon$ term in the introduction’s motivating equation. Revealed preference indifference is observed when irrelevant factors have greater influence, when a judge could be said to have weak preferences over the legally relevant covariates, such as the facts of the case. Another way to benchmark revealed preference indifference is through early predictability, prior to the judge hearing the case.

To illustrate, let me turn to the asylum courts where I have the administrative universe since 1981. This data comprise half a million asylum decisions across 336 hearing locations and 441 judges. These are high stakes decisions whether to deny asylum, which usually results in deportation. The applicant for asylum reasonably fears imprisonment, torture, or death if forced to return to their home country. The average grant rate is about 35%. Chen et al. (2017b) shows that using data only available up to the decision date, you can achieve 80% predictive accuracy. It is predominately driven by trend features and judge characteristics, things that you might wonder if they are unfair, and about one-third is driven by case information, news events, and court information. Then we use only the data available to the case opening and we show that you can achieve 78% accuracy, which raises questions about snap judgments, heuristics, or pre-determined judgments playing a role in how judges decide.

**Fig. 12:**
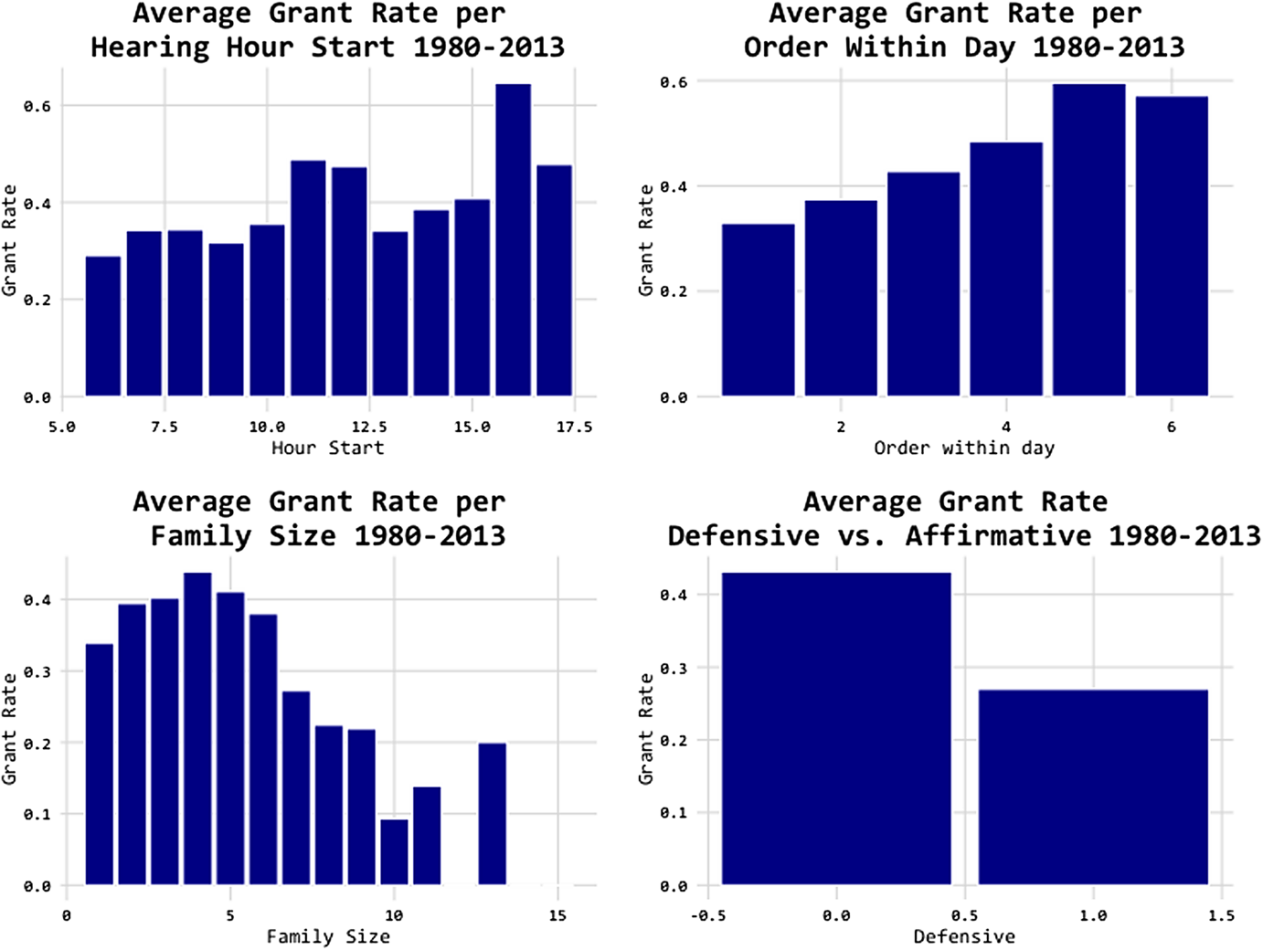
Predictability of asylum decisions (I). *Source*: Chen and Eagel (2017)

Figure 12 shows some descriptive statistics. Judges are more lenient before lunch and towards the end of the day. So this is different in some ways from the Israeli parole article, but otherwise it is consistent in that there are time effects (Danziger et al. 2011). The lower left of this figure shows that there is a U-shape relationship with family size, and the lower right shows that defensive cases are less likely to be granted—defensive cases are those where the applicant has been caught, rather than applying for an extension to stay.

**Fig. 13:**
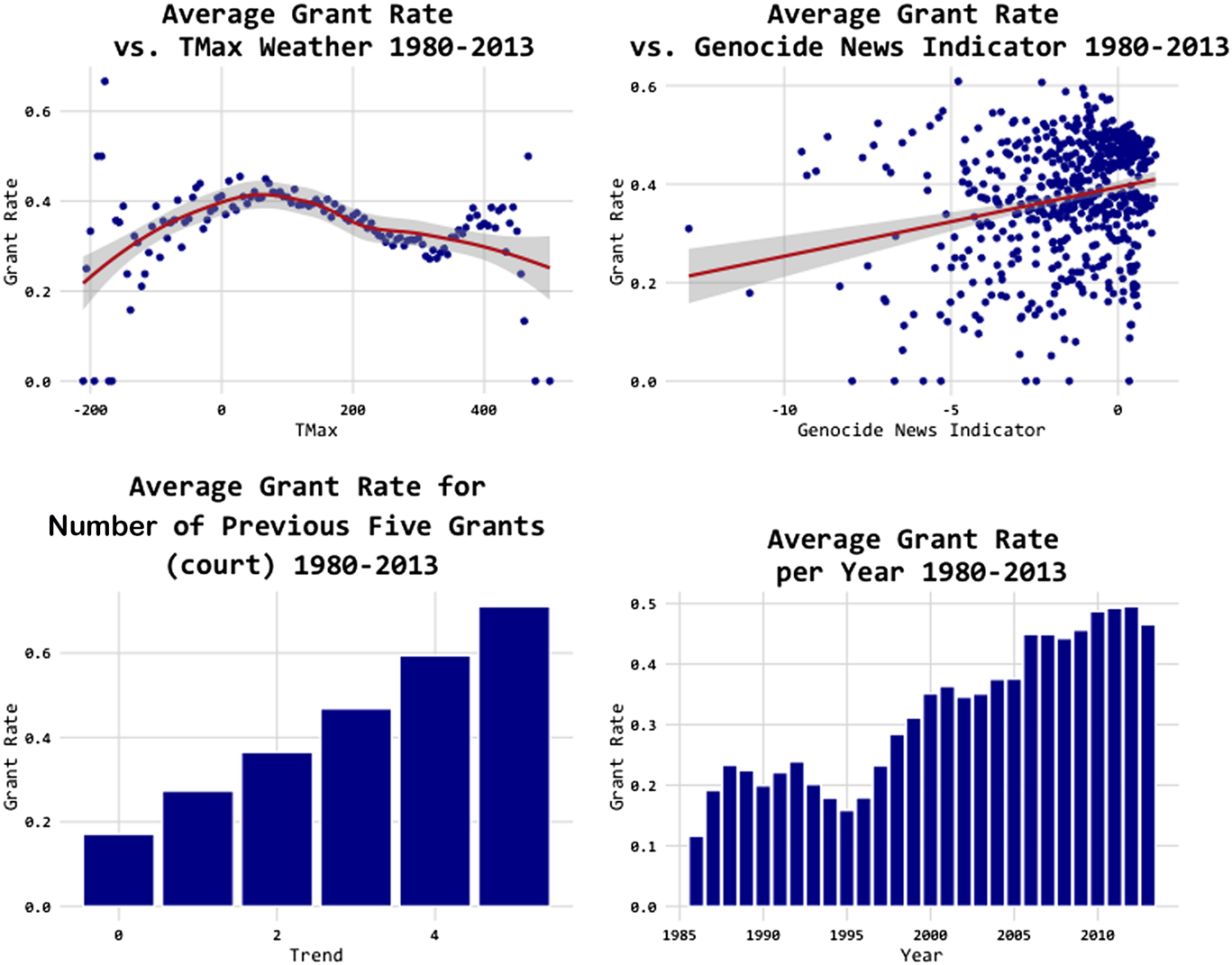
Predictability of asylum decisions (II). *Source*: Chen and Eagel (2017)

Figure 13 shows that judges are more lenient with good weather rather than extreme weather and more lenient with a genocide news indicator. The bottom part shows strong trend factors both within the court on the left and over time on the right. These features are motivated by prior research. For example, Chen (2017) and Heyes and Saberian (2018) also report an effect of temperature and Ramji-Nogales et al. (2007) reports on “refugee roulette”, where the randomly assigned judge has a strong effect on the final decision.

**Fig. 14:**
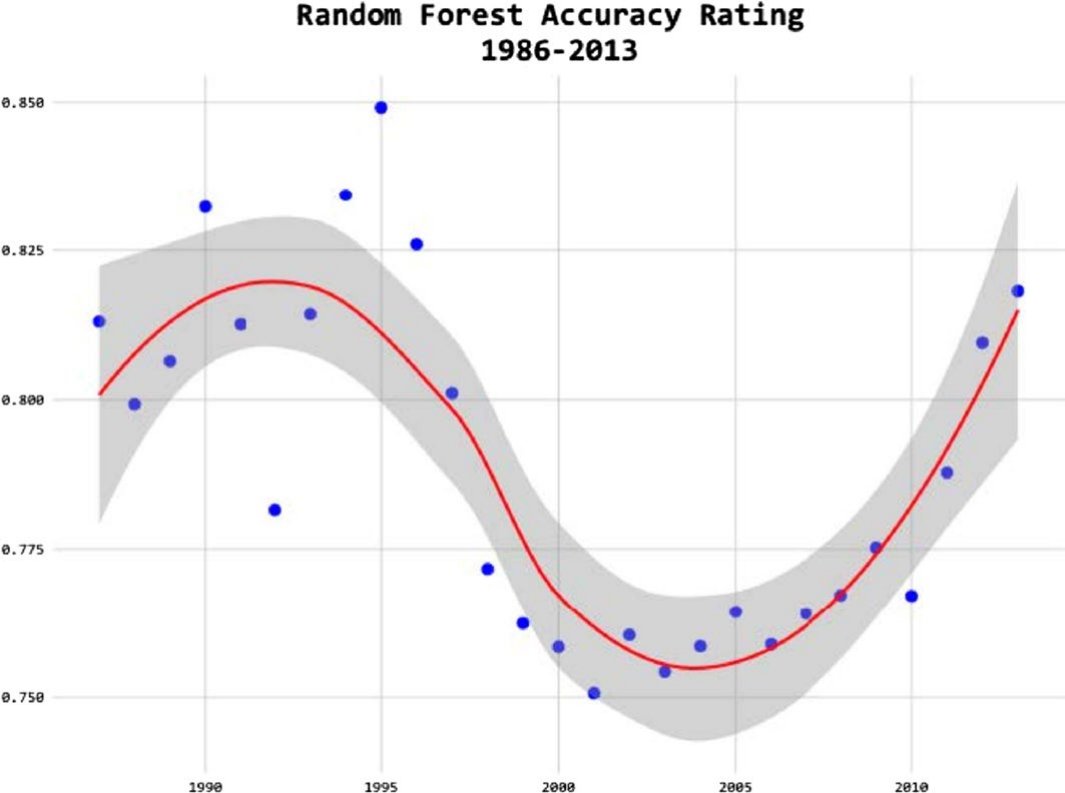
Predictability of asylum decisions. *Source*: Chen and Eagel (2017)

What Chen and Eagel (2017) does is to train a parameter set on all cases up to the preceding December 31st, and it find that random forest performs best. There is a substantial performance dip around the mid-2000s on the test set, as shown in Fig. 14.

**Fig. 15:**
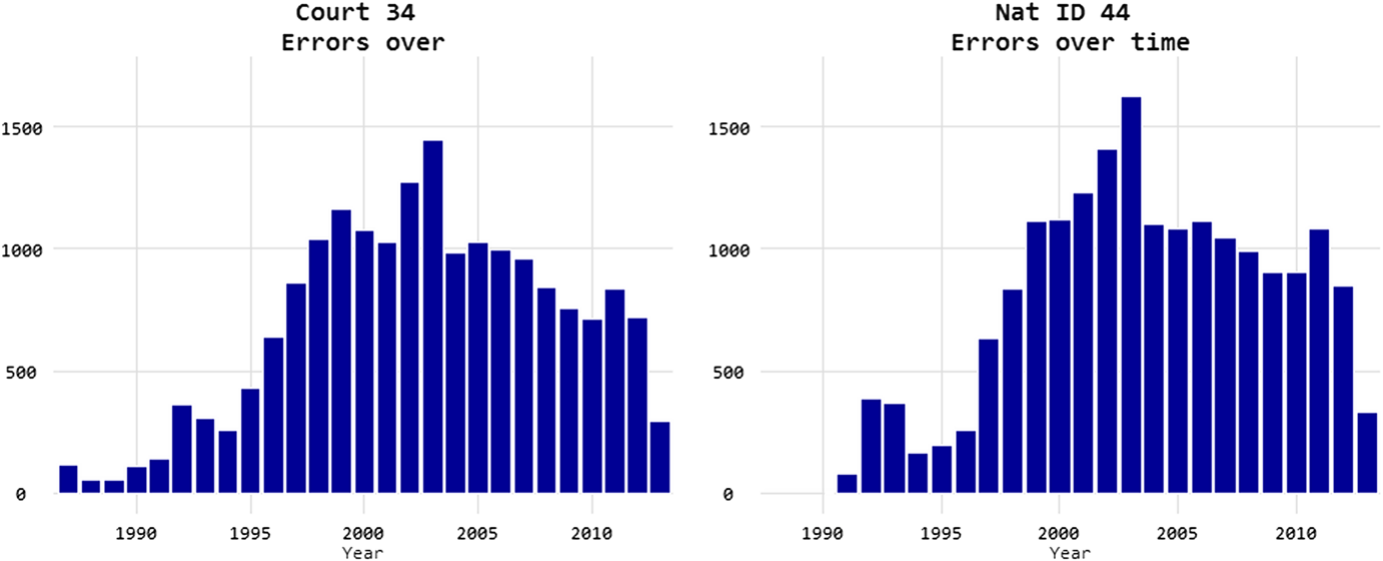
Predictability of asylum decisions. *Source*: Chen and Eagel (2017)

It turns out, with error analysis, 40% of the misclassifications come from Congo applicants in 1 year of city court (and the second Congo war began in 1998 and ended in 2003), as shown in Fig. 15.

Chen et al. (2017b) makes a conceptual distinction between inter-judge disparities in (1) predictions versus (2) prediction accuracy. If case outcomes could be completely predicted after a particular judge is assigned, but prior to judicial inquiry, that would indicate that judges did not take into account any non-coded differences between cases. Now, to be sure, there may be cases in which country and date of application should completely determine outcomes, for example, during violent conflict. But significant inter-judge disparities in predictability would suggest that this understanding of country circumstances does not apply to all judges. Indeed, we find that some judges are highly predictable, always granting or always rejecting, which raises the question of snap judgements or stereotypes—these playing a greater role in decision-making under time pressure and distraction, features that have been articulated to characterize the immigration courts. What we do is to use a minimal set of characteristics: date, nationality, judge, and court (these are, in turn, dummy variables, and motivate using a random forest).

With judge identity we achieve 70% predictive accuracy, and with nationality it is 76% accuracy. Including the opening date, we go from 76 to 78% accuracy. This suggests that variation over time has had little additional impact on the outcome of adjudications. In comparison, with the full model of case completion, we get 82% accuracy. Table 7 reports the accuracy and ROC AUC statistics for the different models.

**Table 7:** Early predictability of asylum decisions

Model	Accuracy	ROC AUC
Judge ID	0.71	0.74
Judge ID and nationality	0.76	0.82
Judge ID and opening date	0.73	0.77
Judge ID and nationality and opening date	0.78	0.84
Full model at case completion	0.82	0.88

**Fig. 16:**
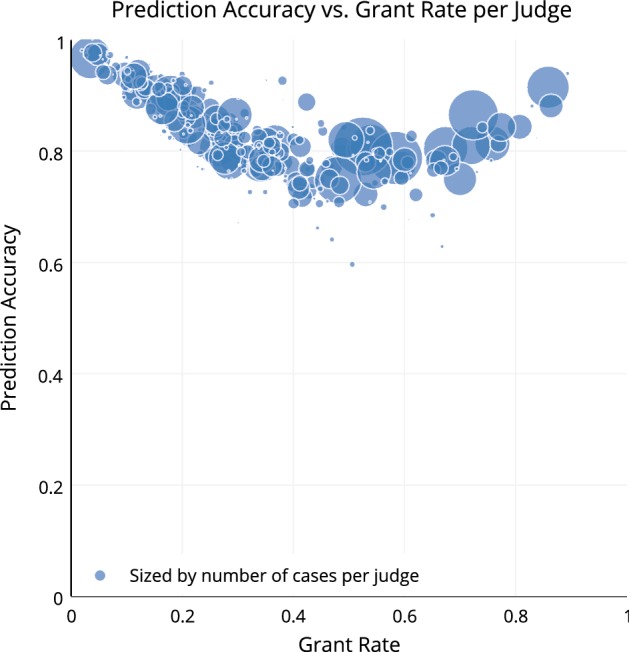
Early predictability of asylum decisions by judge. *Source*: Chen et al. (2017b)

Figure 16 shows that judges with low and high grant rates are more predictable. Each dot represents a judge and the circle size corresponds to the number of cases heard by the judge.

**Fig. 17:**
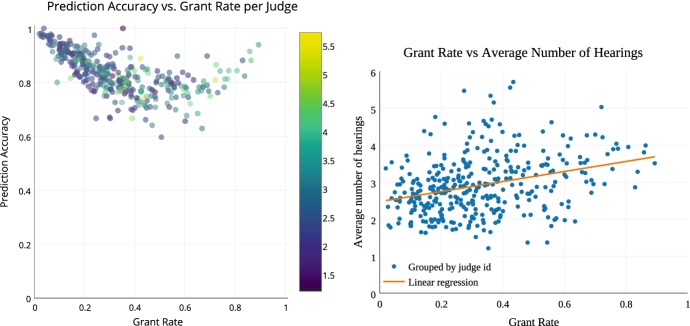
Early predictability of asylum decisions and number of hearings. *Source*: Chen et al. (2017b)

We might wonder, maybe the judges with a middle-grant rate are simply flipping a coin, but that is not the case. Figure 17 shows that middle-grant rate judges hold more hearing sessions than the judges who rarely grant asylum. The color represents the average number of hearing sessions per case.

We may also wonder about the judges that are highly predictable with low or high grant rates—maybe both sides are equally using heuristics. But we see that the judges with higher grant rates are having more hearing sessions on average. It seems that these judges are collecting more information to potentially justify their decisions. Less predictable judges are not simply flipping a coin. Hearing sessions are greater for less predictable judges and for judges with higher grant rates.

## Measuring the consequences of legal precedent

Turning from “what affects judicial decisions” to the question of “what are the effects of judicial decisions”, this section builds on the findings documented in the previous two sections and also on the literature documenting the effects of judge politics, race, and gender (Schanzenbach 2005; Bushway and Piehl 2001; Mustard 2001; Steffensmeier and Demuth 2000; Albonetti 1997; Klein et al. 1978; Humphrey and Fogarty 1987; Thomson and Zingraff 1981; Abrams et al. 2012; Boyd et al. 2010; Shayo and Zussman 2011). A frequent response to findings of extra-legal influences is to debias rulings, perhaps by nudges or through the use of tools from artificial intelligence. This section shows how tools from causal inference can leverage the influence of extra-legal factors to examine the consequences of judicial decisions. Knowledge of these consequences, in turn, may make judges less indifferent to their rulings.

Legal scholars and judges have long made arguments about laws and regulations and justified their arguments with theories about the effects of these legal rules. A particularly challenging dimension of studying the effects of legal rules is that many other aspects of society are correlated with the presence of legal rules, so it is difficult to determine cause or effect. There are judges on the right, such as Judge Richard Posner, who argue that understanding the empirical consequences of judicial decisions is important so that judges can make better cost-benefit utilitarian analyses (Posner 1998). There are judges on the left, such as Justice Stephen Breyer, who also argue that understanding the consequences of their decisions is important so judges can make decisions that accord with the democratic will of the people (Breyer 2006).^3^ Methods to evaluate the impact of court-made law may help judges who are interested in the broader empirical consequences of their decisions.

Consider, for example, a famous Supreme Court case, *Kelo versus City of New London* (2005), where the judges were debating whether to allow government expropriation of private land. The case held that a transfer of private property to another private entity for the purpose of economic development satisfies the public use requirement. The judges debated whether eminent domain would spur economic growth or increase income equality. Justice Ginsburg and Thomas in their dissents argued that taking land from the poor on behalf of a large pharmaceutical company (Pfizer) amounted to “Reverse Robin Hood”. In response to (empirical) policy questions like this, to date, judges speculate on the potential effects of their decisions rather than relying on hard data.

There are three empirical challenges to identifying causal effects. First, legal decisions are not random. They are endogenous to the societal trends that they potentially effect. So how do we determine between cause and effect? Second, there’s substantial cross-fertilization between different legal areas. *Roe versus Wade* (1973) was argued from the part of the law that used to govern government regulation of contracts.^4^ If many legal areas are changing at the same time, how do we know what is the causal effect of one legal area as opposed to another that can be changing at the same time. Third, there’s selection of cases into the courts (Priest and Klein 1984). If the precedent is very strong and in favor of the plaintiff, then weaker cases on the merits may enter into the courts. Plaintiff win rates would reveal little or no information about the underlying strength of precedent.

**Fig. 18:**
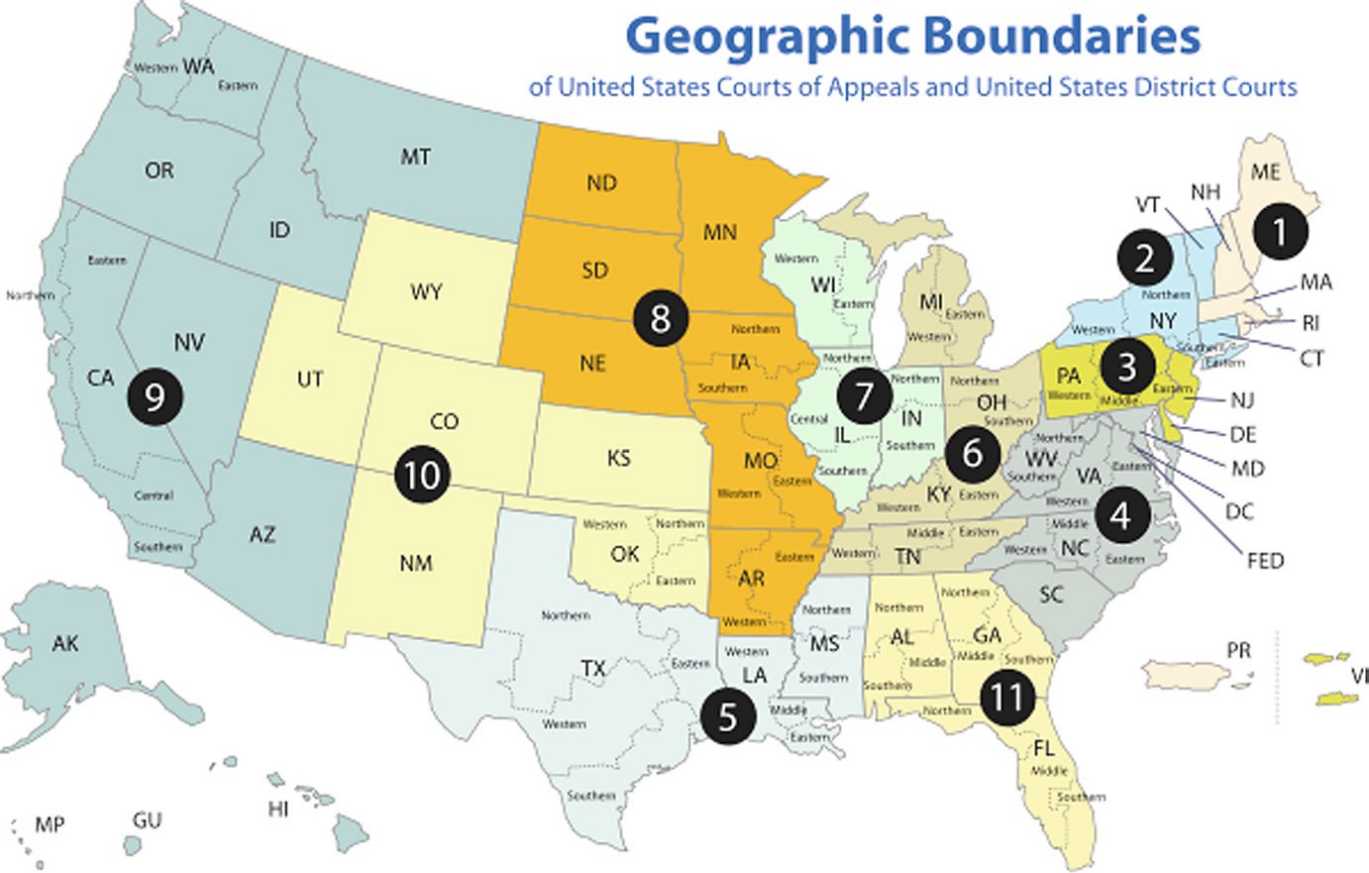
Map of US federal courts

Randomized control trials has also been gaining prominence in economics to evaluate the effects of policies. In law, we cannot randomize judicial decisions, since doing so would undermine the notion of justice and equal treatment before the law, but judges are randomly assigned and there is substantial variation in how they decide—their habits or legal philosophies. For example, Democrats and Republicans decide differently, and this generates a retrospective clinical trial. It was not until a little over 10 years ago when the first article came out that used the random assignment of defendants to harsher or more lenient judges to look at the subsequent outcomes of these defendants over time (Kling 2006). What we can do, then, is to look at the subsequential precedential impacts because the US is a common law system where the case precedent is binding within the Circuits (indicated by the colors in Fig. 18). 98% of the Circuit Court decisions are final. Judges are randomly assigned repeatedly to panels of three, drawn from a pool of 8 to 40 life-tenured judges, who have significant discretion. Their characteristics predict their decisions. Medicine used to also theorize about the effects of medical inventions, but, methods (clinical trials) were developed to evaluate the causal effects of interventions.

More formally, we model the effects of law at the circuit-year (or state-year) level, $Law_{ct}$, on outcome $Y_{ict}$ for individual *i* in circuit *c* at year *t*. The individual could be a person, county, or state—anything that can be aggregated to the circuit level. The second-stage estimating equation is

1 $$\begin{aligned} Y_{ict}=\beta _{0}+\beta _{1}Law_{ct}+\beta _{2}{{\mathbf{1}}}[M_{ct}>0]+\beta _{3}S_{i}+\beta _{4}T_{t}+\beta _{5}X_{ict}+\beta _{6}W_{ct}+\varepsilon _{ict}. \end{aligned}$$

The main coefficient of interest is $\beta _{1}$ on $Law_{ct}$, a measure of the policy direction of decisions issued in Circuit *c* at year *t*. For example, it could be the proportion of “pro-plaintiff” decisions, which is the language we will use here. $M_{ct}$ is the number of cases, $S_{i}$ includes state fixed effects, $T_{t}$ includes time fixed effects, $X_{ict}$ includes state characteristics (such as GDP, population, or state time trends) or individual characteristics (such as gender, age, race, or college attendance), and $W_{ct}$ includes characteristics of the pool of judges available to be assigned.

Let $N_{ct}$ be the number of pro-plaintiff judges assigned to policy-relevant cases. If a circuit-year has a higher fraction of pro-plaintiff judges ($N_{ct}/M_{ct}$) assigned, the precedent for that year will be that much more pro-plaintiff. The moment condition for causal inference is ${{\mathbf{E}}}[(N_{ct}/M_{ct}-{{\mathbf{E}}}(N_{ct}/M_{ct}))\varepsilon _{ict}]=0$, where ${{{{\mathbf{E}}}}}(N_{ct}/M_{ct})$ is the expected proportion of judges who tend to be pro-plaintiff.

The first stage equation is

2 $$\begin{aligned} Law_{ct}=\gamma _{0}+\gamma _{1}Z_{ct}+\gamma _{2}{{\mathbf{1}}}[M_{ct}>0]+\gamma _{3}S_{i}+\gamma _{4}T_{t}+\gamma _{5}X_{ict}+\gamma _{6}W_{ct}+\eta _{ict} \end{aligned}$$

where the terms have been defined as above, and $Z_{ct}$ includes the instruments selected for post-Lasso 2SLS. Estimates for $\mathbf{\gamma }$ and $\mathbf{\beta }$ are estimated using optimal GMM. Standard errors are clustered by circuit-year, since randomization at the circuit-year level addresses serial correlation at the circuit level (Barrios et al. 2012).

Research at the intersection of machine learning and causal inference is moving quickly. One aim of the technology described will be to explicitly allow for future improvements, such that the “engine” can be swapped out, without too much difficulty. For example, recent advances in machine learning and econometrics allow automating the causal analysis of heterogeneous impacts of judicial decisions. Other advances bring deep learning (neural nets) to high-dimensional instrumental variables (such as text), that we can employ to predict the impact of judges’ decisions on populations.

**Fig. 19:**
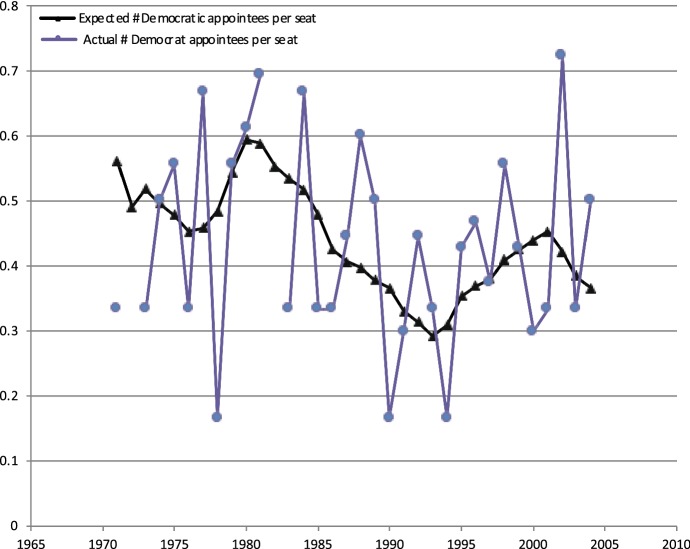
Judicial composition and random assignment. *Source*: Chen et al. (2014)

To illustrate the intuition for our natural experiment, consider Fig. 19. The solid black line is the expected number of Democratic appointees in each seat, which varies systematically over time. The President appoints the judges and the appointments would be correlated with social trends. But the jagged blue line—the actual number of Democratic appointees per seat—varies idiosyncratically around the black line. This idiosyncratic random variation, the jagged blue line, is what we can use to solve the three issues mentioned earlier. First, the randomness would not be caused by future trends. Second, the random variation in one legal area will not be correlated with the random variation in another legal area, which deals with the omitted variables problem. And third, because it is a common law setting, where the precedent is being created through these decisions, the jagged blue line identifies exogenous variation in legal precedent.

The data available to do this kind of analysis comes from hard work from many legal scholars to hand collect cases across a number of different legal areas in civil rights, in property, in constitutional law, to name just a few (Sunstein et al. 2004). Table 8 lists a few. The data is then merged with judge biographies, both from the Federal Judiciary Center as well as separate data collection from newspaper articles (Chen and Yeh 2014a).

**Table 8:** Case categories

Civil rights	Property	Constitutional	Constitutional
Sexual harassment	Eminent domain	Free speech	Abortion
Affirmative action	Corporate veil piercing	Campaign finance	Establishment Clause
Sex discrimination	Contracts	First amendment	Free exercise Clause
Title VII	Environmental protection	Eleventh amendment	Capital punishment
Desegregation	NEPA	Standing	Criminal appeals
Gay rights	Punitive damages	Federalism	
Disability rights	National Labor Review Board	FCC	

**Fig. 20:**
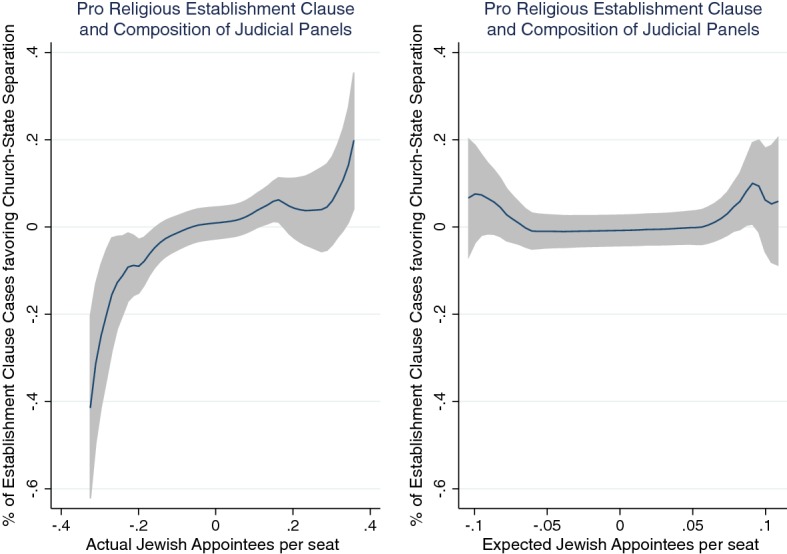
Effect of judge biographies on decisions. *Source*: Ash and Chen (2017)

The correlations between judge biographies and decisions are intuitive. The left side of Fig. 20 illustrates what happens with the Establishment Clause (separation of church and state). This figure plots a local polynomial of the relationship between judicial composition and church-state separation decisions. The shaded areas indicate the confidence intervals. When there are more judges from a minority religion, the more likely they vote to keep church and state separate. The left figure plots the actual Jewish appointees per seat (i.e., the actual composition of the panels assigned to the cases). Whereas on the right, the expected Jewish appointees per seat is not correlated with the precedent (i.e., the expected composition of the panels assigned to the cases).

In an article with econometricians, we show that one does not have to just rely one judicial biographical characteristic (Belloni et al. 2012). There are many characteristics that can be used in a machine learning step to predict the legal decision, as long as the features that are used are all exogenous—that is, from random variation. Moreover, the random variation need not be restricted to biography. It can come from the prior texts or citations by a judge. It can come from extra-legal factors exogenous to the case. Perhaps counter-intuitively, the collection of judicial anomalies in Sect. 2 can be used to measure the causal effects of judicial precedent.

**Fig. 21:**
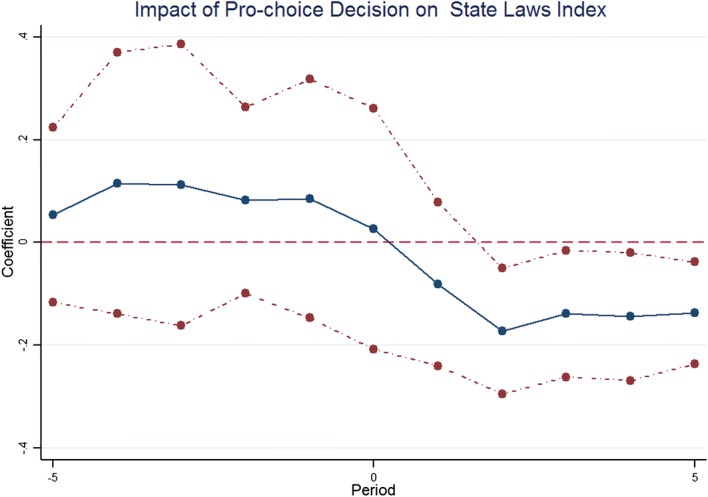
Appellate impact on state laws. *Source*: Chen et al. (2014)

After creating the predictions of decisions, we can look at the effects of the laws on outcomes. For example, Fig. 21 is looking at the effects of pro-choice abortion decisions on state regulations, an index of regulations requiring mandatory delay, banning use of Medicare payments to fund abortion, and requiring parental notification. The solid line indicates the point estimates and the dotted lines the confidence intervals. We can see with a pro-choice abortion decision, states are less likely to have these restrictive laws. It is immediately observed after 1 year, and the pro-choice decision causes an 18% smaller likelihood in each of the regulations in each of the states. Some of this is probably mechanical since the precedent can also be arbitrated over that particular state regulation, but the magnitude would suggest there are also precedential effects. Moreover, there are no lead effects. The state laws are not changing in advance of the Circuit precedent.

In other applications, Chen and Yeh (2014a) examine the effects of government power of eminent domain and finds that it increases economic growth and economic inequality. Chen and Sethi (2011) examine the effects of sexual harassment law and finds that pro-plaintiff decisions increase the adoption of sexual harassment human resources policies and reduce gender inequality in the labor market. Chen and Yeh (2014b) examine the effects of free speech laws and pairs the analysis with an experiment. We can decompose the population effects into both an experimental effect of being directly exposed to the legal change and spillover effects onto those who are not directly affected:

$$\begin{aligned} \rho ={\rm Experimental}\,TOT_{{\rm direct}}\, ^{*} {\rm P}({{\rm exp}}_{\rm direct}) + {\rm Spillovers}\, TOT_{{\rm indirect}}\,^{*}{\rm P}({{\rm exp}}_{{\rm indirect}}) \end{aligned}$$

These example analyses are just the core of a broader analytical and data pipeline that starts from District Court cases, using the random District judge assignment to identify the effect of the presence of an appeal.

- District Cases $$\rightarrow$$
- District Judge Bio $\rightarrow$
- Circuit Case Appeal ${{\mathbf{1}}}[{{\mathrm{M}}}_{\mathrm{ct}}>0]\,\rightarrow$
- Circuit Judge Bio $\rightarrow$
- Circuit Case Decision $Law_{ct}\,\rightarrow$
- Precedential Effects (e.g., State Laws) ${{\rightarrow }}$
- Promulgation (e.g., News) $$\rightarrow$$
- Outcomes

Then, the Circuit judge biographies predict the legal decisions, and these have precedential effects. We can look at the promulgation in newspaper reports and subsequent behavioral outcomes. So far, we have discussed about pro versus anti decisions, but we can also use the presence of a case to consider pro versus no case versus anti. What I mean is we can flip a coin, and it can be heads or tails, but we can also wonder what happens when we did not have a coin flip at all. To put it differently, we might wonder what society would be like had *Roe versus Wade *been decided the opposite way, or what society would be like if *Roe versus Wade *did not exist as an event.

Now let me discuss briefly on modularity and extensibility. The pipeline above comes from the laborious hand collection, but one might want to automate the Chicago Judges Project (Sunstein et al. 2004). For example, a District Court case comes up to the Circuit Court, and we might want to automatically identify the nearest case. Also, instead of relying on many years of law students’ hand coding the direction of the case, we can do fast-decision classification. In a different direction, we might broaden the question to not just whether there is an effect of the decision—affirm or reverse—but look at the text itself: Does the dicta matter? Does the reasoning or citation matter? Could the document embedding or other low-dimensional representation of judicial opinions be used to characterize a set of policy levers? What about creating deep predictions from the judicial corpora of how the judges have previously decided? The potential steps could be as follows: (1) train word2vec, (2) form document embeddings, (3) use deep IV to identify the direction in the embedding space that is causally related to societal changes, (4) form k-means clusters in the word2vec space, and (5) report phrases in the same cluster that are far away from each other along the predictive dimension. These steps are illustrated in other articles.

## Conclusion

Let me end with a note on other prediction projects that employ the 12 terabytes of collected data. For example, in Supreme Court studies, the benchmark explanatory models include political ideology and historical voting trends, but we can incorporate the Circuit Court text, the oral argument text, the audio files, and lawyer biographies. We can also study the Supreme Court Justices own writings prior to appointment to the Supreme Court. Through the published decisions of all 26 appellate judges who sat on at least fifty circuit cases and later served on the Supreme Court from 1946 to 2016, Ash and Chen (2018) find that a judge who moves from the most Democrat to the most Republican in precedent and phrase usage is 32% points and 23% points, respectively, more likely to vote conservative. A judge who moves from the lowest to highest rank in similarity to Richard Posner and in economics usage is 18% points and 6% points, respectively, more likely to vote conservative. A judge who moves from the lowest to highest rank in vote polarization and electoral dissent is 25% points and 8% points, respectively, more likely to vote conservative. We can also predict reversals, not just going from Circuit to Supreme Court, but also from the District to Circuit Courts. A recent study by Caliskan et al. (2017) showed that word embeddings of the Stanford Internet Corpus reflect human-like semantic biases. What we can do is to look at the judge’s own past writings and see if that correlates with their biographies, and when the judges are randomly assigned, does it impact the decisions? Does it predict sentencing harshness and disparities? Ash et al. (2018) shows that economic thinking of judges is strongly predictive of sentencing harshness. The idea that if legal institutions can not catch suspects, then the judge might increase the sanctions so the expected deterrence is the same.

We can also try to predict re-arrest and prosecutor screening decisions using a unique dataset followed from the police arrest report. An algorithm would reduce the re-arrest rates for a set charge rate. We also find that prosecutors seem to be releasing defendants throughout the risk distribution. We can also predict ideology, in particular the political donations of the Supreme Court lawyers, using both their text and their audio. The audio doubles predictive accuracy relative to the text alone. Motivated by the error analysis that found the Congo war to vastly help with predictions, we digitized the Wikileak cables data to predict the asylum grants and claims. Finally, we can quantitatively assess the oft-stated story that judges, on the record, go so far as to say that they changed the facts as described from the District Court fact descriptions to justify a legal change. One of the things we are trying to do is to identify the fact section versus the legal section, and then characterize judicial fact discretion, and see if this is predictive of the reversals of lower court decisions as well as the subsequent response to these judges.

The legal profession is undergoing a great transformation. The tools of machine learning and causal inference can be used to study, predict, and respond to normative judgments. In this article, I discuss how these tools can assess extra-legal factors that predict judicial decisions and how these predictions can be used to measure the causal impacts of judicial decisions.

## Footnotes

1 After the great transformation, human economic mentalities were changed, and people became more economically rational, behaving as neoclassical economic theory would predict (Polanyi 1944).

2 US Courts of Appeals Database Project (http://www.cas.sc.edu/poli/juri/auburndata.htm).

3 Judge Richard Posner has lamented that, “[judicial] opinions lack the empirical support that is crucial to sound constitutional adjudication” (Posner 1998); similarly Justice Breyer remarked, “I believe that a[n] interpretive approach that undervalues consequences, by undervaluing related constitutional objectives, exacts a constitutional price that is too high” (Breyer 2006).

4 *Roe versus Wade *extended the right of privacy under the Due Process Clause of the 14th Amendment. Even though the Due Process Clause was previously interpreted as precluding government interference in freedom of contract (see, e.g., *Allgeyer versus Louisiana* (1897)), it was then interpreted as precluding interference in a woman’s decision to have an abortion.
